# Spice Production, Marketing, and Value Chain in Ethiopia

**DOI:** 10.1155/2024/5211327

**Published:** 2024-01-23

**Authors:** Abdurahman Wondimnew

**Affiliations:** Department of Biology, College of Natural and Computational Sciences, Mekdela Amba University, P.O. Box 32, Tulu Awuliya, Ethiopia

## Abstract

The purpose of this paper was to document and organize spice production, marketing, and value chain in Ethiopia. Spice is the most essential farming product. Several native and exotic spices are grown by smallholder farmers across the country because Ethiopia has diverse agroecology and favorable climate conditions. The spice subsector has great potential for the country's economic development and poverty alleviation, cultivation, preparation, transport, and merchandising of spices and herbs. Spice market indicates the commodity value of spices. The major problems regarding the marketing system of spices were lack of communication between farmers and users, lack of linkage between sellers and buyers, and lack of postharvest management, storage facilities, regulated and cooperative markets, transportation, and knowledge of market news. The escalating value of spices is superficially envisioned, extending from smallholder production to partial handling, which seems to be the everyday value chain.

## 1. Introduction

“Any aromatic vegetable substance in the whole, broken, or ground form which is used primarily to season food rather than to contribute nutrients” is what the United States Food and Drug Administration (FDA) defines as a spice (as cited in [1], para. 4). Spices are the most profitable venture in all farming activities as they provide ample employment opportunities and slope to raise the income of the farming community. Ethiopia has a long history of spices. It is a country that ships many spices with the normal yield secured by the spice being around 222,700 hectares with a production of 244,000 tons per year. Ethiopia supplies over 50 spices, with an estimated 200,000 hectares potentially providing more spices [2].

Ethiopia may be a country for many spices, such as korarima (*Aframomum corrorima*), long red pepper, dark cumin, white cumin/ministers weed, coriander, fenugreek, turmeric, sage, cinnamon, and ginger [3]. The different agroclimatic environment in Ethiopia underpins developing a wide assortment of crops in common and spice crops in particular. In this way, the nation has a few indigenous common and intriguing spice crops, which have been cultivated widely since time immemorial. Spice crops are produced in different districts of the nation and overwhelmingly by smallholder agriculturists as cash trim exchanged essentially in domestic markets, but with expanding victory also entering remote markets. The spices subsector has an immense potential for financial advancement and poverty decrease through creation and development of employment openings and conveyance of wage and foreign trade earnings. However, pepper (*Capsicum annum*) and spice crop cultivation is conventional, with nothing or exceptionally little.

In Ethiopia, the production and use of aromas are perhaps the most important sentiment in the legendary botanical accounts dating back to the days of the rulers of Sheba. Korarima cardamom, Ethiopian long pepper, black cumin, bishop herb, and coriander flavoring plants such as thyme and fenugreek have also been thought to be associated with Ethiopia as roots or centers of difference. Ethiopian spices are also many, and the role they played can be seen in comparison to their level of use and they have been used as one of the main occupations and life-giving foods, offering a rare opportunity to accelerate both rural and urban improvements [4].

Ethiopia has a wide range of encounters in spice production. Asfaw and Tadesse [5] state that the Ethiopian people have long used and still use flavors, spices, additives, and herbs within the framework of their traditional diet; Ethiopian kitchen gardens are located in backyards, front yards, side yards, or mostly surround homes. There are plenty of crops with climate, water resources, and soil suitable for growing spices; in addition, there is an appropriate investment approach which facilitates long-range adjustment investments through payroll tax exemptions, equipment and equipment follow-up tax exemptions, and loss carry forward credits.

Value chain analysis has become an important tool for studying agricultural markets in the creation of economies as it enables a better understanding of production strategies, supply phases, presentations, and dynamics between screen characters in the chain [6]. It has the potential to recognize intercessions that benefit the poorest and least efficient actors [7]. This study identifies the connections between performing artists, practices, and screen characters within the Korarima value chain and recognizes the main constraints. Intermediation possibilities are discussed, and the challenges and dilemmas of assessing these in terms of their impact on poverty, gender, and environment (PEG) are, so to speak, not related to the particular spice, but to overall considerations.

## 2. Main Text

### 2.1. Definition of Spices

As stated by Goshme [8], any dried plant product, including seeds, leaves, bark/peel, and flowers, that is used primarily for flavoring is referred to as a spice in other ways. Spices are essential oils that impart flavor, aroma, and occasionally color to meals and beverages. They can be sold wholly as a powdered form or as oleoresins and essential oils. There are numerous other uses for spices; others, like peppers (Capsicum spp.), serve as alternatives to chemical dyes or pesticides, while plants like turmeric (*Curcuma longa*) are becoming more and more popular for use as natural remedies. According to another definition, a spice is a dried seed, fruit, root, bark, or vegetative substance that is added to food in nutritionally small amounts for flavoring and occasionally as a preservative by eradicating or stopping the development of dangerous microorganisms. In accordance with Helman [9], spices are diverse parts of plants, including bark, flowers, roots, seeds, nuts, and natural products, derived from known plants. It does not contain and is used as condiments, flavors, and food flavors (dried or fresh excrement is considered an herb and is therefore not flavored). However, spices and herbs have long been most commonly used to enhance the tactile properties of foods because they contain compounds with interesting flavor properties [10].

### 2.2. Production of Spices in Ethiopia

The total spice production in Ethiopia increased from 234,000 tons in 2013 to 356,000 tons in 2018, while the area under spice cultivation increased from 150,000 ha to 207,000 ha. Chilli pepper is the most commonly produced spice, accounting for over 80% of total production and exports, and ginger was the second most produced spice crop until 2013. Kifelew et al. [11] estimated that black cumin, ajwain or bishop's weed, fenugreek, and coriander had 36 and 17% share in area and production, respectively. In relation to Hibistu [12], the average annual growth rate of spices was 25.6 and 25.3% in volume and values between 1998 and 2010.

Dessalegn [13] asserts that spices play a significant role in Ethiopian cuisine. Since the beginning of time, Ethiopia has produced and consumed spices and is one of Africa's biggest consumers of spices. Spices are used to flavor meats, soups, vegetables, bread, butter, and other foods. Additionally, they produce medications and perfumes with spices [14]. Many spices, including korarima (*Aframomnum corrorima*), long red pepper, black cumin, white cumin, coriander, fenugreek, turmeric, sage, cinnamon, and ginger, are native to Ethiopia [14].

Masresha [15] claims that Ethiopia's complex agroecology encourages the growth of a wide variety of crops in general and spice crops in particular. As a result, the nation is home to several indigenous common and exotic spice crops, which have been widely farmed since the dawn of time. The majority of the country's smallholder farmers grow spice crops, which are mostly traded in domestic markets but are also increasingly making inroads into overseas markets. Through the establishment and extension of employment opportunities, as well as the distribution of income and foreign exchange revenues, the spice subsector has a significant potential to contribute to economic growth and the elimination of poverty.

As plant species, tastes have a high likelihood of being developed in various agrobiological zones around the country, claims ACP [16]; but the growth of pepper (*Capsicum annuum* L.) spices is traditional; no advances have been achieved in seed or planting material; the development process and methodology heavily rely on knowledge that has been passed down from generation to generation. Many smallholders utilize flavors to flavor fabrics, as a source of basic oil, as a source of color, and as a source of income; flavors are one of Ethiopia's traditional high-value agricultural crops. According to Herms [17], Ethiopia has a remarkable capacity to increase taste production, largely because of its good climate, unending arable land, inexpensive labor, proximity to potential markets, and welcoming approach environment. Natural conditions that are favorable and enticing selections of lowland and fine country flavors have been released for consumers; therefore, it could be a very good occasion to expand and disseminate the current advancements inside the nation's proposed agroecologies [18]. In relation to Mathewos [19], the interchange of flavors on a national and international scale brings in money for producer ranches, retailers involved in the distribution network, and the nation as a whole.

Spices are developed completely from different agrobiological zones of the nation. Accessibility of money-related administrations by banks and microcredit teach furthermore global attractiveness of spice crops has a high potential for development and enhancement of trade profit of Ethiopia, motivations enables exporters to implement modern processing methods and equipment, spanning from preassembly to post-assembly processing. A growing number of buyers, traders, oleoresin extraction companies, and pharmaceutical producers select to purchase flavors from Ethiopia [20].

In accordance with Tesfa et al. [21], to empower private speculation, the Ethiopian government has created a bundle of motivating forces beneath control No. 84/2003 for speculators locked in unused endeavors and developments, over a run of segments. These motivating forces are accessible both to remote and household speculators and the directions do not segregate between outside and domestic investors or foreign investors of different nationalities.

According to Herms [17], the zone beneath flavor cultivation in Ethiopia shifts every year and spatially, for instance, it has been between 330,000 ha and 500,000 ha in the period 2005–2013. Adding up to flavor generation expanded from 238,000 million tons in 2005 to 418,000 million tons in 2013. Chilies, ginger, dark cumin, dark cardamom, and turmeric accounted for 97% of the national annual normal flavor generation volume within the same period. In Ethiopia, the Southern National and Nationalities People Groups Territorial States (SNNP) is the locale which produces the greatest number of spices in the country.

### 2.3. Global Evidence of Spice Market

Marketing is the method for bringing sellers and buyers together for the purpose of exchanging product names and administrations [22]. The global spice trade has continued to grow over the years. For instance, this amount increased eightfold, reaching 38 billion USD in 2015 compared to 5 billion USD in 1991; this tendency was mostly caused by population growth (in 1991, it was only 5,000 million USD), 3 billion people on the planet; in 2015, this number was nearly 7.5 billion.

This increase essentially follows the increase in all products, including agricultural products, and also shows a clear upward trend, with the growth rate of overall merchandise trade being faster. This trend is mainly explained by the increase in commodity production which is generally higher than agricultural and food production. Further significant reductions in transport and transaction costs also play a role in this regard [23].

### 2.4. African Spice Market

Although the use of most African-produced spices is still limited to the continent, this is the beginning to change. In 2013, the intercontinental spice trade accounted for about 23% of the total traded value of spices; however, by 2019, that percentage had dropped to just 11%.

From 1.58 million metric tons in 2013 to 1.62 million metric tons in 2018 (and reaching a peak of 1.78 million metric tons in 2017), Africa became a major producer of spices. The proportion of African production in the world market has dropped from 15% to 13%. This is mostly because, according to FAOSTAT, spice production has increased more in other regions, particularly in Asia and Latin America.

### 2.5. Ethiopian Spice Market

Today, Ethiopia is one of the largest consumers of spices in Africa. Ethiopia grows many spices, which are used not only to flavor bread, butter, meats, soups, and vegetables but also to produce medicines and perfumes. Most of the spices produced (96%) are consumed domestically. According to Kotler and Keller [24], promoting is characterized as a social and administrative handle whereby people and bunches get what they require and need through making and trading items and esteeming others. The American Promoting Association representing promoting experts within the US and Canada states that marketing is the method for arranging and executing the conception, estimating, advancement, dispersion of thoughts, merchandise, and administrations to form trades that fulfill person and organizational destinations [25].

According to Kaplinsky and Morris [26], the value chain describes the full range of activities which are required to bring a product or service from conception, through the different phases of production (involving a combination of physical transformation and the input of various producer services), delivery to final consumers, and final disposal after use.

**Figure 1:**
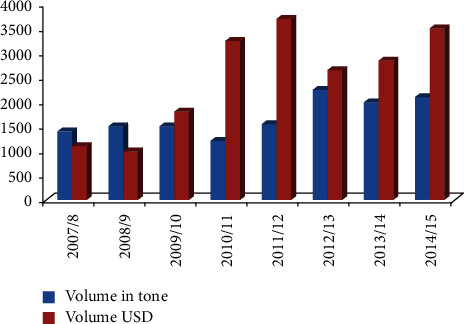
Export performance of spice.

In spite of the reality that Ethiopia is being a country of various spice crops and favorable agroecologies and long history of flavor generation, so distant the nation is not recognized as a major exporter of flavors and, consequently, the share of flavors of the country's add up to trade profit is at high level (Ethiopian Service of Industry), 2015 ENTAG (Ethiopia–Netherlands Exchange) [17]. Internationally, there are more than 109 varieties of spices that are traded commercially (ENTAG (Ethiopia–Netherlands Trade for Agricultural Growth), 2018). Ethiopia exports ginger, pepper (crushed or ground), turmeric, coriander, cumin, fenugreek, cardamom, cinnamon and clove (EMI (Ethiopian Ministry of Industry), 2015). Export trend (tons) for different spice crops in Ethiopia is shown in Figure 1.

Generally speaking, the nation's total trade profit [27], from spice exports has stayed below 1%, but for a considerable amount of time in 2003/04 and 2004/05, the profit of spice exports increased to 1.1% and 1.4%, respectively. During trimming a long time from 1997/98 to 2009/10, the aggregate normal development rates of trade of flavors were 25.6 and 25.3% in volume and esteem, individually Yimer and Demirli [27].

According to Yimer and Demirli [27], the country earned $11,128 million and $18,568 million, respectively, for two consecutive years from 2008/09 to 2009/10 Worldwide Exchange Middle [28]. Excluding cardamom, fenugreek, pepper, and other spice products, the sales success rates for flavors in the 2009/10 launch year were 8.4%, 64.9%, and 15.4%, respectively. In 2013 and 2014, the spice trade was worth 15,000 tons a year, worth $26 million. Ginger is the most exported peel this year, accounting for about 50% of the total trade value (Ethiopia–Netherlands Exchange for Rural Development), 2018 [17]. Ginger showed full flavor with 60.75% volume over the long term from 2007/08 to 2012/13, followed by turmeric (11.47%), pepper (9.26%), and cumin (8.85%) paddy field (Ethiopian Industrial Services (EMI), 2014). Furthermore, until 2013, ginger was the most traded flavor in Ethiopia, with a trade value of US$12 million in 2013, which could account for 45% of the trade value [17, 29]. Chili peppers soon followed, followed by turmeric and dark cumin flavors in 2013 (EMI (Ethiopian Industrial Service), 2015; ENTAG (Ethiopian–Dutch Exchange for Agricultural Development), 2018).

According to Aveling et al. [30]. many of his NTFPs (nontimber forest products, including spices) are harvested as a livelihood and source of income for many rural households in Ethiopia, although most commodities are sold at local or street markets. According to Bekele and Pillai [31], markets continue to be seen as a means of ensuring that small producers of agricultural commodities are effectively integrated into the mainstream economy, especially in developing countries [32]. Conversely, poor market performance threatens the sustainability of production and makes it difficult to continuously supply raw materials to the market.

### 2.6. Export Destinations of Ethiopian Spices

Although the majority of Ethiopian spices exported go to other African nations, the export market is changing. Between 2013 and 2017, the proportion of Ethiopian spice exports to African nations fell from 61% to 53%. Information for 2019 and 2018 is not accessible.

In 2017, 40% of the entire value of exports came from Sudan. The United States, Egypt, and Djibouti are further significant export markets. Spice shipments to Asia are rising: between 2013 and 2017, the proportion of exports rose from 15% to 24%. 10% of it arrived in Europe in 2017 as opposed to 4% in 2013, a 63% rise in absolute terms. This will continue to go on as the European market for spices continues to expand. Spice exports to the Middle East dropped by 53 percent between 2013 and 2017. When considering all of Ethiopia's exports, the countries that trade with China most frequently are Somalia, the United States, Saudi Arabia, Germany, and the Netherlands.

### 2.7. Spice Value Chain in Ethiopia

In relation to Kaplinsky and Morris [33], they define the value chain as manufacturer, delivery to the end user, and final disposal after use. Most definitions of value chains use “chain” to indicate the vertical relationship between producers and buyers (chain actors) and the movement of a particular source or product from the producer to consumer.

In relation to Rieple and Singh [34], a typical value chain analysis breaks down the stages of a product from production to market to identify inefficiencies or areas of inefficiency. Most analyzes look at ways to address these issues and share profits more fairly among actors in the chain.

According to Rieple and Singh [34], value chain analysis has been applied to many sectors, from agricultural products to garment production, and has been described as an “adaptive model” due to its popularity and versatility. However, the main criticism of value chain diagnostic work is that many studies are top-down and the limited involvement of key stakeholders [35].

This is illustrated by the Ethiopian Spice Strategy [16]; this strategy was developed as a result of just one participatory stakeholder workshop; in relation to [36, 37], it did not reach the recommended level of cooperation with growers and harvesters.

**Figure 2:**
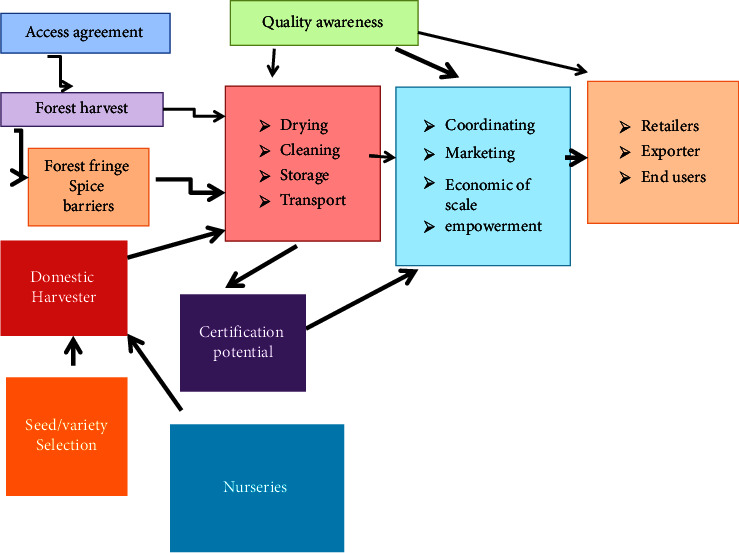
Spice value chain in Ethiopia.

According to CSA [38], preparing a spice supply in Ethiopia involves a number of activities such as supply of inputs, seed/variety selection, nursery and exchange plot arrangements, seed propagation, delivery arrangements, sowing/crop production, cultivating safety, weed and weed control, collecting, beating, semidrying/complete drying and application of suitable drying strategies, washing of foreign substances/mixtures, proper pressing and use of suitable pressing materials, collection/replenishment of collected fragrances, staging, wholesalers, processors, and manufacturing plants (oleoresin milling/processing (including mills), retail and home consumption, and shipping to foreign markets (Figure 2).

This is illustrated by Tiru et al. [39] that challenges start from the need for awareness on spice generation, handling, capacity, and promotion among makers. Ranchers are still utilizing their traditional cultivating hones and ordinarily collect exceptionally low yields. So, also, the promoting framework is not managed through organized endeavors. The price of flavors is not decided by the request, supply, and cost data but rather by person choice. A person choice making handle of the promotion of spice leads to wasteful and ineffective benefit of the advertise. This suggests that agriculturists are not getting anticipated benefits from this division. Weak regulatory education, lacking transportation, control and water framework, and the nonappearance of important upstream value chain performing artists, such as hardware, seed, and fertilizer providers, and firms providing supporting services are moreover challenges to be tended to [40]. This is illustrated by Mathewos [19] that the spice value chain in Ethiopia is strong to outside stuns and, with that, its capacity to proceed conveyed a sustainable supply to meet requests.

Key actors within the spice value chain in Ethiopia incorporate agriculturists and collectors at the cultivate door, input providers (seeds, fertilizer, bundling, and transport), agents/brokers who act as a medium between collectors and European clients, dealers who work as vital, and taking ownership of the item and processors [41]. Although research on spice show profit and the value chain there are still numerous issues that need to be resolved by the segment. For instance, the nonappearance of insufficiency improves technologies, postharvest dealing with, unpredictable supply and variable quality of flavors delivered from woodland and agricultural landscapes, destitute showcase and interfacing street framework in major spice creating zones, and the need for cash crop orientation towards tall esteemed globally exchanged crops [17, 19, 42–44].

## 3. Opportunity and Constraints of Spice Production and Marketing in Ethiopia

- Ethiopia has diversified agroecology and favorable climatic conditions for the production of spices
- The spice subsector has enormous potential to reduce poverty in Ethiopia, create and develop commercial possibilities, and generate money for the farming community as a cash crop [21, 45]
- Ethiopia is making significant investments in agroindustrial parks in an effort to attract investors, commercialize production, and encourage local value addition
- It is possible to intercrop spices with other well-established crops that have an effective value chain, like coffee
- Spices that have been processed are far more expensive than those that are raw. In this regard, rural value addition offers investors great chances to boost margins
- Low production of spices due to farmers using traditional farming practices
- Lack of knowledge about spice production, processing, storage, and marketing information among producers and consumers
- The marketing system is not handled through concerted efforts
- The farmer did not profit from the spice industry because of a lack of value chin.

## 4. Conclusion

Ethiopia may be a source of distinctive spices with different major agroecological zones and different agroecological subzones. Ethiopia incorporates a reasonable climate for cultivating different types of spices. The nation produces numerous spices which are exchanged as export things. Spices are used to flavor diverse nourishment and to form medicines and fragrances. Within the nation, there is a need of mindfulness on flavor generation, preparing, capacity, and marketing among makers. Ranchers utilized their conventional cultivating hones and more often than not gathered very low abdicate. Generation and promotion of flavors are influenced by different variables. To move forward the production and promoting the potential of the nation, satisfying different inputs at profitable price, introducing unused advances, mindfulness on pre- and postharvest flavor management, constructing diverse foundations, creating coordinates that bother and illness management, interventions within the coordination of flavors promoting exercises and arrangement of advertise support services, expanding esteem expansion of flavors and take after cost-effective promoting channels, developing good showcasing investigates and making advancements, expanding government back, and others are crucial. The spice sector has immense potential for economic development and poverty reduction. Value chain analysis is an important tool for studying agricultural products and market analysis in the creating economies for a better understanding of production strategies and supply phases.

## 5. Recommendation

Spices are one of the key subsectors that fit into the commercialization of agriculture plan as a cash crop. Due to their cash crop status, spices have a significant potential to increase smallholder farmers' purchasing power, hence reducing poverty and guaranteeing food security. However, there has not been much funding or attention from research and extension for the production of spice crops. It is therefore important to note that policymakers, researchers, and extension agencies should take more action in support of spice crops throughout the world and in Ethiopia specifically. Farmers cultivate a variety of spices, including fenugreek, capsicum, black and white cumin, coriander, and basil; research and extension could initiate intervention on these spices. Spice production would gain value from the collection, characterization, and analysis of essential oils and other key constituents, thereby enhancing the livelihoods of farmers through the use of spices. To determine value chain players and limitations, a thorough analysis of the spice value chain is required. For farmers to receive better prices, strong market ties between retailers, wholesalers, and consumers must be formed. The goal of the review should be to increase the productivity and production of spices by creating better agronomic techniques and improved varieties and to motivate marketing networks between the farmer and consumers and to enhance the economic growth of the country.

## Data Availability

All data used in this study are available within the article.
